# Endophthalmitis rates after macular hole and epiretinal membrane surgery: a multicentre TriNetX study

**DOI:** 10.1038/s41433-026-04700-0

**Published:** 2026-08-03

**Authors:** Sonny Caplash, Joseph Wahba, Brian L. VanderBeek

**Affiliations:** 1 Scheie Eye Institute, Department of Ophthalmology, University of Pennsylvania Perelman School of Medicine, Philadelphia, PA USA; 2 Center for Pharmacoepidemiology Research and Training, University of Pennsylvania Perelman School of Medicine, Philadelphia, PA USA; 3 Leonard Davis Institute, University of Pennsylvania Perelman School of Medicine, Philadelphia, PA USA

**Keywords:** Risk factors, Retinal diseases

## Abstract

**Purpose:**

Previous work has postulated that a post-vitrectomy gas bubble disrupts capillary action between the external eye and vitreous cavity, thereby reducing the risk of endophthalmitis. To test this theory, we will investigate the rates of endophthalmitis between macular hole surgery versus membrane peeling for epiretinal membrane.

**Methods:**

Data was extracted from TriNetX, a de-identified global electronic health record database. Patients were queried for specific Current Procedural Terminology (CPT) codes 67041 and 67042, and International Classification of Disease (ICD) 10 codes H35.34x (Macular hole) and H35.37x (macular puckering/epiretinal membrane). Outcome was defined as any diagnosis of endophthalmitis (H44.0x) within 14 days of initial surgery. Propensity score matching (PSM) was performed to balance baseline demographic and clinical characteristics. Patients were excluded if they had any prior history of endophthalmitis.

**Results:**

23 490 eligible epiretinal membrane (ERM) surgeries and 15 346 eligible macular hole (MH) surgeries were included. After PSM, 14 545 matched surgeries in each cohort were analysed. In the ERM group, there were a total of 52 patients who developed endophthalmitis. In the MH group, there were 20. This yielded a risk ratio of 2.60 (95% CI: 1.55, 4.35; p-value of 0.000).

**Conclusion:**

Pars plana vitrectomy for ERM appears to have a higher risk for subsequent endophthalmitis as compared to vitrectomy for MH. This study is limited by an inability to control for vitrectomy gauge and confirm use of gas or air tamponade.

## Introduction

Exogenous endophthalmitis is a severe, vision-threatening complication of intraocular surgery. A recent large-scale study over a 22 year period estimated the rate of endophthalmitis after retina surgery to be 0.16%, with an overall downward trend over time [1]. While the decrease in endophthalmitis rate is likely multifactorial, one surgical aspect theorised to reduce bacterial transmission has been the use of gas tamponade in post-vitrectomised eyes [2, 3].

This assertion is supported by two separate studies that have noted that the majority of collected endophthalmitis cases across the literature occurred in fluid-filled as opposed to gas-filled eyes [4, 5]. The first study to postulate this association was a two-centre study that analysed early rates of endophthalmitis after the introduction of 23- and 25-guage vitrectomies as physicians acclimated to the smaller gauged vitrectomies [3]. A more recent single-centre study examined 26 cases of endophthalmitis after vitrectomies across all indications and found that fluid-filled eyes had higher rates of endophthalmitis [6]. A French nationwide database study also demonstrated similar results [7]. Taken together, these offer early evidence that gas tamponade may play a role in reducing rates of endophthalmitis.

To test this hypothesis, we will use a large international federated health network database to compare the endophthalmitis rate between two very similar types of vitrectomy surgery: epiretinal membrane peeling and macular hole repair. The major difference between the surgeries is the use of gas tamponade.

## Methods

Data was extracted from TriNetX, a de-identified global electronic health record database of roughly 200 million patients from 160 healthcare organisations (HCOs) across 21 countries. The database contains diagnoses, procedures, medications, laboratory values, and genomic information from a diverse population of patients ranging from the Americas and Europe to the Middle East, Africa, and the Asia-Pacific. Due to the de-identified nature of the data, the University of Pennsylvania’s Institutional Review Board deemed this study exempt from review. The Strobe checklist for observational studies was followed throughout this study.

Patients were queried for specific Current Procedural Terminology (CPT) codes 67041 and 67042 and International Classification of Disease (ICD) 10 codes H35.34x (Macular hole) and H35.37x (macular puckering/epiretinal membrane). Supplemental Fig. 1 illustrates query criteria for Macular puckering (Cohort 1) and macular hole (Cohort 2). The index event was defined as the date of surgery.

Propensity score matching (PSM) was performed prior to running outcome analysis to balance baseline demographic and clinical characteristics such as hypertension, diabetes mellitus, history of intravitreal injections, smoking, and corticosteroid use. Patients were excluded if they had any prior history of endophthalmitis, a history of uveitis or less than 3 months of follow up were excluded. The primary outcome was defined as any diagnosis of endophthalmitis (H44.0x) within 14 days of initial surgery. Risk ratios were then calculated between the endophthalmitis rate after ERM and macular hole surgeries. TriNet X has statistical software built into its analytic database which was used for the conduction of this study.

## Results

23 490 eligible epiretinal membrane (ERM) surgeries and 15 346 eligible macular hole (MH) surgeries were gathered from the initial query of the TriNetX database. After PSM, 14 545 matched surgeries in each cohort were balanced for baseline demographic and clinical characteristics. The demographic breakdown after PSM matching for the ERM cohort was 67.0 (SD ± 10.9) years old, 62.3% Female, and 73.5% White. The demographic breakdown for the MH cohort after PSM matching was 66.9 (10.7) years old, 62.0% Female and 73.6% White. After PSM matching of clinical characteristics, the ERM and MH cohorts were found to be similar in rates of diabetes mellitus (ERM = 27.7%, MH = 27.5%), hypertension (ERM 58.4%, MH = 58.2%), history of corticosteroid use (ERM = 2.7%, MH = 2.7%), tobacco use (ERM = 1.5%, MH = 1.6%) and history of intravitreal injection (ERM = 4.5%, MH = 4.4%). Please see Table 1 for a detailed summary of baseline characteristics before and after PSM matching.

**Table 1:** Baseline demographics before and after propensity score matching.

Covariate	Cohort	Mean (± SD)	Patients	% of Cohort	Standardised Mean Difference	Mean (± SD)	Patients	% of Cohort	Standardised Mean Difference
Age at Index	ERM Mac Hole	66.9 (11.4) 67.0 (10.8)	23,490 15,346	100% 100%	0.020	67.0 (10.9) 66.90 (10.7)	14,545 14,545	100% 100%	0.003
Female	ERM Mac Hole		11,823 9,757	50.3% 63.6%	0.270		9,061 9,018	62.3% 62.0%	0.006
White	ERM Mac Hole		17,854 10,729	76.0% 69.9%	0.137		10,692 10,711	73.5% 73.6%	0.003
Black or African American	ERM Mac Hole		1,998 2,255	8.5% 14.7%	0.194		1,646 1,635	11.3% 11.2%	0.002
American Indian or Alaska Native	ERM Mac Hole		65 38	0.3% 0.2%	0.006		38 37	0.3% 0.3%	0.001
Asian	ERM Mac Hole		1,201 611	5.1% 4.0%	0.054		613 611	4.2% 4.2%	0.001
Native Hawaiian or Other Pacific Islander	ERM Mac Hole		94 48	0.4% 0.3%	0.015		41 48	0.3% 0.3%	0.009
Other Race	ERM Mac Hole		814 582	3.5% 3.8%	0.017		543 534	3.7% 3.7%	0.003
Unknown Race	ERM Mac Hole		1,464 1,083	6.2% 7.1%	0.033		972 969	6.7% 6.7%	0.001
**Diagnosis**									
Diabetes mellitus	ERM Mac Hole		7,519 4,046	32.0% 26.4%	0.124		4,022 3,999	27.7% 27.5%	0.004
Hypertensive diseases	ERM Mac Hole		14,210 8,950	60.5% 58.3%	0.044		8,492 8,461	58.4% 58.2%	0.004
Long term (current) use of steroids	ERM Mac Hole		743 414	3.2% 2.7%	0.028		398 397	2.7% 2.7%	<0.001
Tobacco use	ERM Mac Hole		366 270	1.6% 1.8%	0.016		219 239	1.5% 1.6%	0.011
**Procedure**									
Surgical Procedures on the Conjunctiva	ERM Mac Hole		869 453	3.7% 3.0%	0.042		421 445	2.9% 3.1%	0.010
Surgical Procedures on the Anterior Segment of the Eye	ERM Mac Hole		7,821 3,432	33.3% 22.4%	0.246		3,409 3,385	23.4% 23.3%	0.004
Surgical Procedures on the Ocular Adnexa	ERM Mac Hole		1,009 389	4.3% 2.5%	0.097		399 389	2.7% 2.7%	0.004
Intravitreal injection of a pharmacologic agent (separate procedure)	ERM Mac Hole		2,294 646	9.8% 4.2%	0.219		659 646	4.5% 4.4%	0.004

The total rate of endophthalmitis among all patients after PSM was 0.24%. In the ERM group, 52 patients developed endophthalmitis, yielding a rate of 0.36%. In the MH group, 20 patients developed endophthalmitis, yielding a rate of 0.14%. This yielded an increased risk for endophthalmitis within the ERM cohort, with a risk ratio of 2.60 (95% CI: 1.55, 4.35; p-value of 0.000). The absolute risk difference was 0.22% for a number needed to harm of 454 surgeries.

A sensitivity analysis was performed to investigate the risk ratio for endophthalmitis at 7 days and 42 days, which demonstrated a similar trend of increased endophthalmitis rates in the ERM cohort compared to MH. Risk ratios were similar among all 3 time points. Complete results from the sensitivity analysis are detailed in Table 2.

**Table 2:** Risk Ratio analysis results.

Follow-up period (Days)	Cohort	Patients in cohort	Endophthalmitis (# of patients)	Risk	Risk ratio (95%CI)
7	ERM	14,545	45	0.003	3.00 (1.67–5.38)
	Macular Hole	14,545	15	0.001	
14	ERM	14,545	58	0.004	2.60 (1.55–4.35)
	Macular Hole	14,545	24	0.002	
42	ERM	14,545	60	0.004	2.38 (1.46–3.67)
	Macular Hole	14,545	26	0.002	

## Discussion

This study evaluated the rate of post-surgical endophthalmitis between epiretinal membrane surgery with macular hole surgery. Our results reveal an increased risk of endophthalmitis in patients undergoing epiretinal membrane surgery. The risk difference of 0.22% represents one additional case of endophthalmitis for every 454 vitrectomies performed for ERMs compared with MH. For context, the absolute reduction of 0.22% found in our study may not seem large; however, it is roughly equal to the absolute reduction in post-intravitreal injection endophthalmitis associated with the use of betadine [8, 9].

This study validates the work of previous publications from regional surgical centres that had smaller sample sizes [6, 10]. Our results are also confirmatory of a previous large, nationwide French database study in which the incident rate ratio of endophthalmitis for ERM surgery compared with that of macular hole surgery was 2.24, a very similar outcome to our rate ratio of 2.60 [7]. Procedurally, ERM surgery and macular hole surgery are similar in location of pathology, degree of vitrectomy performed, and additional additive surgical manoeuvres such as ERM and internal limiting membrane (ILM) peeling. Their principal difference is in gas tamponade at the end of the case. The majority of retina surgeons in the United States and internationally use gas tamponade in macular hole surgery [11], and in epiretinal membrane surgery, patients are often left with either fluid or air tamponade [12]. This study design allows for two cohorts, with the principal variable being the presence of gas tamponade. Additionally, the larger sample size provides adequate power to reliably explore this hypothesis. It is important to note, as well, that the queried HCOs are international, which adds to the generalisability of our results. This study is consistent with previous studies examining the rate of endophthalmitis after vitrectomy for MH or ERM, adding to its external validity [7].

Gas tamponade has previously been postulated to allow for better wound closure in sutureless vitrectomy. Work by Yamane et al. has validated stronger wound closure with gas tamponade using anterior-segment OCT [13]. Better wound closure allows less microbial ingress into the eye. Gas filled eyes have also been hypothesised to have lower rates of endophthalmitis because of disrupted capillary action. This more specifically refers to vitreous wicks which can act as a conduit for microbial infection. This mechanism has been postulated as a cause of endophthalmitis following intracapsular cataract surgery [14, 15] and pars plana vitrectomy [3]. With gas tamponade, there is a theoretical disruption of this conduit which may contribute to lower endophthalmitis rates. Additionally, gas tamponade reduces the incidence of hypotony, which can theoretically act as a driving force for infectious agents. These hypotheses have been postulated and supported previously, especially in work that compared the rates of endophthalmitis between sutured 20 G vitrectomy and sutureless vitrectomy [2, 3, 16, 17].

Several limitations to this study need to be noted. First, patients are selected using ICD and CPT codes, which cannot be independently verified, nor can culture results be obtained, but disease coding has proven reliable for many ophthalmic conditions [18]. Next, while it is unlikely that significant differences exist in the vitrectomy-gauge between the two cohorts, we are unable to control for the gauge of vitrectomy used during surgery and cannot definitively rule out the possibility that this impacted the outcomes. Nor are we able to account for which surgeries had their wounds sutured and what type of gas tamponade was used. In addition, it is possible that some of the ERM surgeries ended with an air-fluid exchange, which would make the ERM cohort like the MH cohort. While this misclassification may have occurred, this type of bias would bias to the null and suggests the actual risk difference between the two groups would be larger than stated here. Also of note, for an unmeasured confounding variable to have produced the results we found here, it would have had to had a risk ratio of 4.6 (e-value), which is unlikely given how few factors are known to impact the rate of endophthalmitis. Lastly, we are unable to account for ocular factors that may have impacted the rate of endophthalmitis (e.g. pseudophakia) or post-op drop compliance.

This study suggests an association between gas tamponade and reduced rates of endophthalmitis with gas tamponade. The degree of this effect is relatively small with an absolute risk ratio of 0.22%. Given the inability to correct for a variety of surgery-specific factors, it is important to note that this study implies an association and does not prove any causation. Further work, such as a prospective study that controls for the gauge of vitrectomy incisions and accounts for sutured wounds, is needed to establish more definitive causation.

## Summary

### What was known before

- Previous, smaller sample studies hypothesised that gas tamponade may reduce risk of endophthalmitis.
- This hypothesis was extrapolated from studies that compared endophthalmitis rates between different gauge vitrectomy systems by looking at a secondary analysis of gas versus fluid filled eyes.
- Data were extracted from North America primarily.

### What this study adds

- Leveraging data from a de-identified global electronic health record database of roughly 200 million patients, we evaluated the rates of endophthalmitis after macular hole surgery against epsiretinal membrane surgery.

- These comparators were chosen as their principal difference is often the use of gas tamponade.
- This study found a nearly 50% lower rate of endophthalmitis after macular hole surgery compared with epiretinal membrane surgery.
- To our knowledge, this is the largest scale study exploring this clinical question and advances our understanding of endophthalmitis and the role of gas tamponade.

## Supplementary information

Supplemental Figure 1

Supplemental figure 2

## Data Availability

All data are freely available to anyone at any institution that provides data to the TriNet X database, but by contract cannot be distributed beyond those at qualifying institutions.

## References

[1] VanderBeek BL, Chen Y, Tomaiuolo M, Deaner J, Syed Z, Achyara B, et al. Endophthalmitis rates and types of treatments after intraocular procedures. JAMA Ophthalmol. 2024;142:827–34. 10.1001/jamaophthalmol.2024.2749. PMC11295066 39088207

[2] Scott IU, Flynn HW Jr., Dev S, Shaikh S, Mittra R, Arevalo JF, et al. Endophthalmitis after 25-gauge and 20-gauge pars plana vitrectomy: incidence and outcomes. Retina. 2008;28:138–42. 10.1097/IAE.0b013e31815e9313. 18185150

[3] Scott IU, Flynn HW Jr., Dev S, Shaikh S, Mittra R, Arevalo JF, et al. Incidence of endophthalmitis after 20-gauge vs 23-gauge vs 25-gauge pars plana vitrectomy. Graefes Arch Clin Exp Ophthalmol. 2011;249:377–80. 10.1007/s00417-010-1505-8. 20853005

[4] Bahrani HM, Fazelat AA, Thomas M, Hirose T, Kroll AJ, Lou P, et al. Endophthalmitis in the era of small gauge transconjunctival sutureless vitrectomy–meta analysis and review of literature. Seminars in ophthalmology. 2010;25:275–82. 10.3109/08820538.2010.518109. 21091012

[5] Chen G, Tzekov R, Li W, Jiang F, Mao S, Tong Y. INCIDENCE OF ENDOPHTHALMITIS AFTER VITRECTOMY: A Systematic Review and Meta-analysis. Retina. 2019;39:844–52. 10.1097/IAE.0000000000002055. 29370034

[6] Miller CW, Rabljenovic A, Papproth C, Sciulli Md H, Platt Md S, Miller DG Characteristics and Risks of Endophthalmitis after 25-gauge Vitrectomy Surgery over a 14-year Period. Ophthalmol Retina. 2024. 10.1016/j.oret.2024.11.019. 39617213

[7] Ben Ghezala I, Mariet AS, Benzenine E, Bron A, Baudin F, Daien V, et al. Incidence of acute postoperative endophthalmitis following macular surgery in France between 2006 and 2016. Acta Ophthalmol. 2020;98:e333–8. 10.1111/aos.14279. 31680475

[8] Speaker MG, Menikoff JA. Prophylaxis of endophthalmitis with topical povidone-iodine. Ophthalmology. 1991;98:1769–75. 10.1016/s0161-6420(91)32052-9. 1775308

[9] Trinavarat A, Atchaneeyasakul LO, Nopmaneejumruslers C, Inson K. Reduction of endophthalmitis rate after cataract surgery with preoperative 5% povidone-iodine. Dermatology. 2006;212:35–40. 10.1159/000089197. 16490973

[10] Chiang A, Kaiser RS, Avery RL, Duge P, Eliott D, Shah S, et al. Endophthalmitis in microincision vitrectomy: outcomes of gas-filled eyes. Retina. 2011;31:1513–7. 10.1097/IAE.0b013e3182209290. 21878799

[11] *2024 Membership Survey: Preferences and Trends*. 2024. https://www.asrs.org/content/documents/_asrs-2024-pat-survey-for-website.pdf.

[12] Okamoto F, Okamoto Y, Hiraoka T, Oshika T. Effect of vitrectomy for epiretinal membrane on visual function and vision-related quality of life. Am J Ophthalmol. 2009;147:869–74, 874 e1. 10.1016/j.ajo.2008.11.018. 19200531

[13] Yamane S, Kadonosono K, Inoue M, Kobayashi S, Watanabe Y, Arakawa A. Effect of intravitreal gas tamponade for sutureless vitrectomy wounds: three-dimensional corneal and anterior segment optical coherence tomography study. Retina. 2011;31:702–6. 10.1097/IAE.0b013e3181f0d2e6. 21242862

[14] Maxwell DP Jr., Diamond JG, May DR. Surgical wound defects associated with endophthalmitis. Ophthalmic Surg. 1994;25:157–61. 8196919

[15] Lindstrom RL, Doughman DJ. Bacterial endophthalmitis associated with vitreous wick. Ann Ophthalmol. 1979;11:1775–8. 318047

[16] Shimada H, Nakashizuka H, Hattori T, Mori R, Mizutani Y, Yuzawa M. Incidence of endophthalmitis after 20- and 25-gauge vitrectomy causes and prevention. Ophthalmology. 2008;115:2215–20. 10.1016/j.ophtha.2008.07.015. 18930557

[17] Kunimoto DY, Kaiser RS. Wills Eye Retina Service. Incidence of endophthalmitis after 20- and 25-gauge vitrectomy. Ophthalmology. 2007;114:2133–7. 10.1016/j.ophtha.2007.08.009. 17916378

[18] Cai CX, Michalak SM, Stinnett SS, Muir KW, Fekrat S, Borkar DS. Effect of ICD-9 to ICD-10 Transition on Accuracy of Codes for Stage of Diabetic Retinopathy and Related Complications: Results from the CODER Study. Ophthalmol Retina. 2021;5:374–80. 10.1016/j.oret.2020.08.004. 32810681

