# Supplementary material for: Endophthalmitis rates after macular hole and epiretinal membrane surgery: a multicentre TriNetX study
Source: Eye (Lond). 2026 Aug 3;40(15):2355–9. doi: 10.1038/s41433-026-04700-0 (PMC13675174; doi:10.1038/s41433-026-04700-0)
Supplement: Supplementary file 1 [file 41433_2026_4700_MOESM1_ESM.pdf]

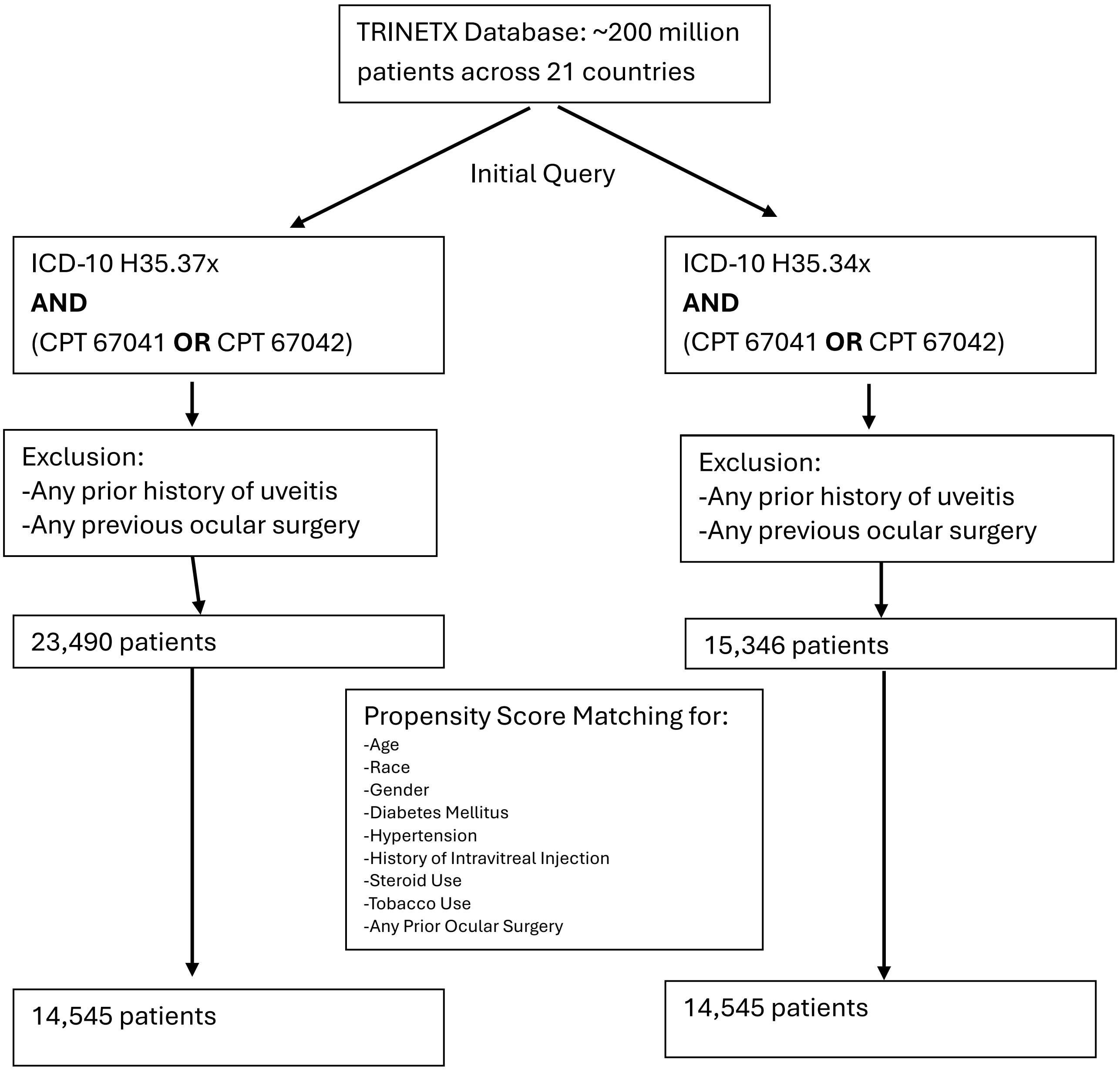
TRINETX Database: ~200 million patients across 21 countries
Initial Query
ICD-10 H35.37x
AND
(CPT 67041 OR CPT 67042)
ICD-10 H35.34x
AND
(CPT 67041 OR CPT 67042)
Exclusion:
-Any prior history of uveitis
-Any previous ocular surgery
Exclusion:
-Any prior history of uveitis
-Any previous ocular surgery
23,490 patients
15,346 patients
Propensity Score Matching for:
-Age
-Race
-Gender
-Diabetes Mellitus
-Hypertension
-History of Intravitreal Injection
-Steroid Use
-Tobacco Use
-Any Prior Ocular Surgery
14,545 patients
14,545 patients
