# Supplementary material for: Endophthalmitis rates after macular hole and epiretinal membrane surgery: a multicentre TriNetX study
Source: Eye (Lond). 2026 Aug 3;40(15):2355–9. doi: 10.1038/s41433-026-04700-0 (PMC13675174; doi:10.1038/s41433-026-04700-0)

Supplemental figure 2: Propensity Score Plots (ERM - purple, Mac Hole – green)

A) Before propensity score matching:

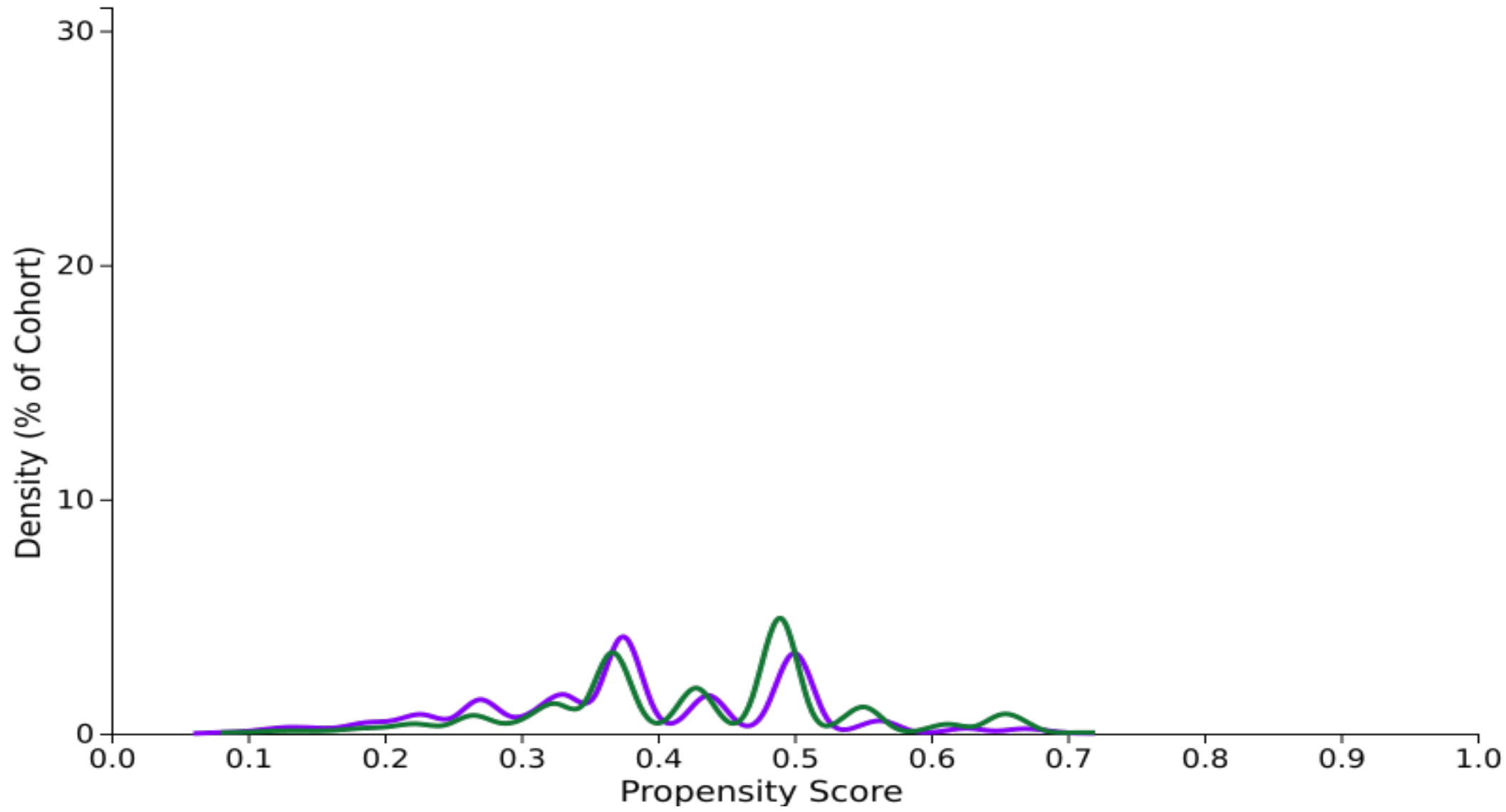

B) After propensity score matching:

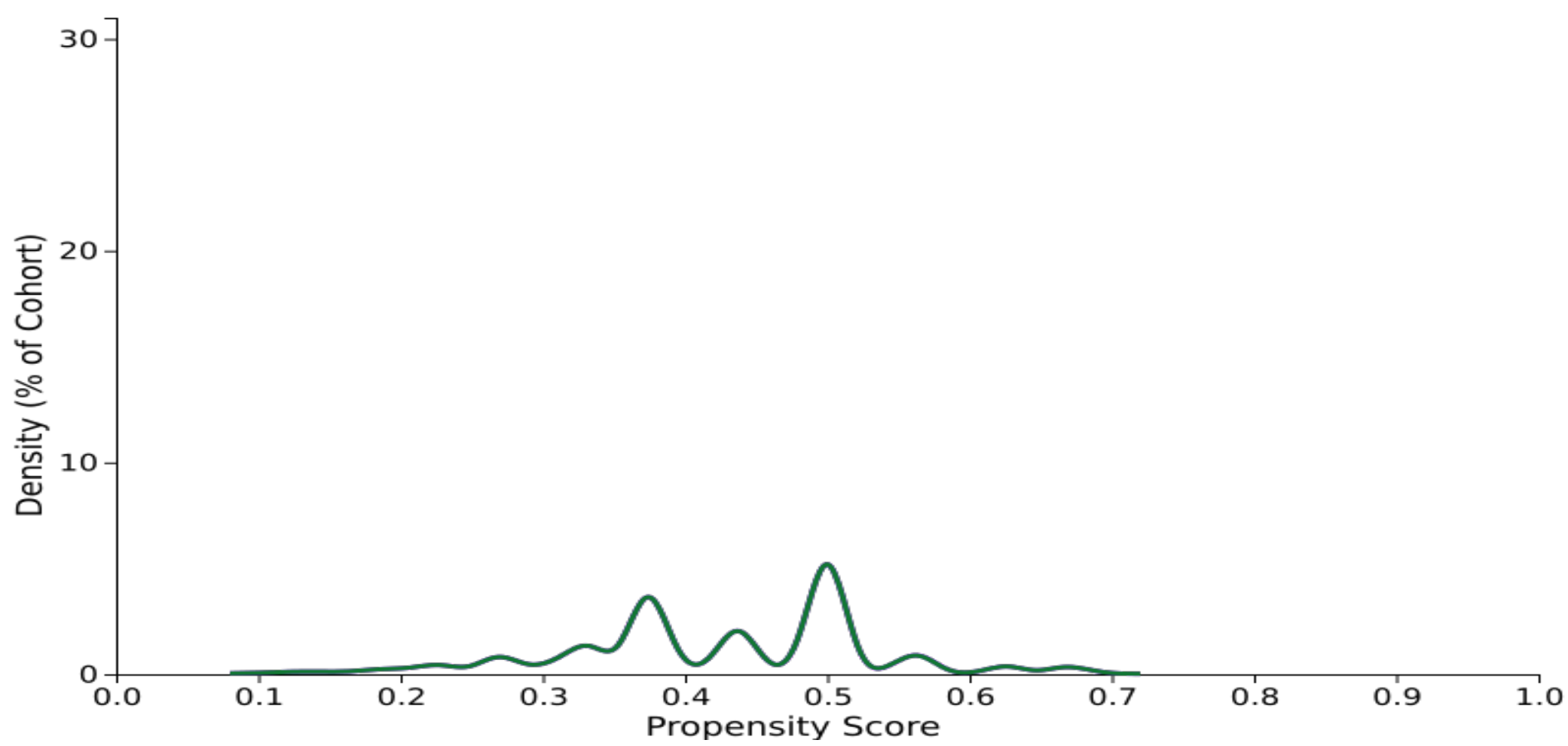

Supplement: Supplementary file 2 [file 41433_2026_4700_MOESM2_ESM.pdf]
